# Cerebral Microdialysis-Based Interventions Targeting Delayed Cerebral Ischemia Following Aneurysmal Subarachnoid Hemorrhage

**DOI:** 10.1007/s12028-022-01492-5

**Published:** 2022-04-29

**Authors:** Jakob Winberg, Isabella Holm, David Cederberg, Malin Rundgren, Erik Kronvall, Niklas Marklund

**Affiliations:** 1 Department of Clinical Sciences Lund, Lund University, Lund, Sweden; 2 Department of Clinical Sciences Lund, Lund University, Skåne University Hospital, Neurosurgery, EA-blocket plan 4, Entrégatan 7, 222 42 Lund, Sweden

**Keywords:** Cerebral microdialysis, Subarachnoid hemorrhage, Delayed cerebral ischemia, Clinical application

## Abstract

**Background:**

Delayed cerebral ischemia (DCI), a complication of subarachnoid hemorrhage (SAH), is linked to cerebral vasospasm and associated with poor long-term outcome. We implemented a structured cerebral microdialysis (CMD) based protocol using the lactate/pyruvate ratio (LPR) as an indicator of the cerebral energy metabolic status in the neurocritical care decision making, using an LPR ≥ 30 as a cutoff suggesting an energy metabolic disturbance. We hypothesized that CMD monitoring could contribute to active, protocol-driven therapeutic interventions that may lead to the improved management of patients with SAH.

**Methods:**

Between 2018 and 2020, 49 invasively monitored patients with SAH, median Glasgow Coma Scale 11 (range 3–15), and World Federation of Neurosurgical Societies scale 4 (range 1–5) on admission receiving CMD were included. We defined a major CMD event as an LPR ≥ 40 for ≥ 2 h and a minor CMD event as an LPR ≥ 30 for ≥ 2 h.

**Results:**

We analyzed 7,223 CMD samples over a median of 6 days (5–8). Eight patients had no CMD events. In 41 patients, 113 minor events were recorded, and in 23 patients 42 major events were recorded. Our local protocols were adhered to in 40 major (95%) and 98 minor events (87%), with an active intervention in 32 (76%) and 71 (63%), respectively. Normalization of energy metabolic status (defined as four consecutive samples with LPR < 30 for minor and LPR < 40 for major events) was seen after 69% of major and 59% of minor events. The incidence of DCI-related infarcts was 10% (five patients), with only two observed in a CMD-monitored brain region.

**Conclusions:**

Active interventions were initiated in a majority of LPR events based on CMD monitoring. A low DCI incidence was observed, which may be associated with the active interventions. The potential aid of CMD in the clinical decision-making targeting DCI needs confirmation in additional SAH studies.

**Supplementary Information:**

The online version contains supplementary material available at 10.1007/s12028-022-01492-5.

## Introduction

Aneurysmal subarachnoid hemorrhage (SAH) accounts for 5% of all strokes and has a peak incidence between 50 and 60 years of age [1, 2]. Despite rapid neurosurgical management, including treatment of the ruptured aneurysm, morbidity and mortality remains high [3, 4]. Common complications include cerebral vasospasm and delayed cerebral ischemia (DCI), affecting approximately one third of patients with SAH. DCI may manifest as a clinical deterioration, either as a decline in the level of consciousness or through the emergence, or as an aggravation, of focal neurological deficits [5, 6]. DCI may also lead to the development of cerebral infarcts, and DCI-related infarcts are a main contributor to poor long-term outcome in patients with SAH [6, 7]. Although the pathophysiology remains unclear, complex events that include neuroinflammation, excitotoxicity and microvasospasm as well as macrovasospasm contribute to the development of DCI [8–10]. While large vessel vasospasm and DCI are separate entities, verified vasospasm is still considered a key contributor to the development of DCI [11, 12]. The only established pharmacological prophylaxis is the calcium channel antagonist nimodipine. Despite lacking robust evidence for preventing vasospasm, nimodipine has demonstrated neuroprotective effects and to reduce the onset of new neurological deficits with improved neurological outcome in patients with SAH [13]. Neurocritical care (NCC) management of vasospasm include preventing—and correcting—hyponatremia, hyperglycemia, fever, and hypoxia and maintaining normovolemia [14–18]. Optimal hemoglobin (Hb) concentration in patients with SAH is debated. However, a Hb threshold of > 8–10 g/dL was advocated in the latest recommendations from the Neurocritical Care Society’s Multidisciplinary Consensus Conference [19]. Induced hypertension to increase cerebral blood flow, as well as endovascular angioplasty, are also widely accepted in vasospasm treatment [20, 21].

The detection of DCI is complex, especially in patients with poor-grade SAH, as they often require sedation and mechanical ventilation. Thereby, advanced multimodal neuromonitoring in the NCC unit is required to adjust cerebral and systemic physiology to prevent the development of manifest cerebral infarcts [22, 23]. Besides routine intracranial pressure (ICP) and cerebral perfusion pressure (CPP) monitoring, frequent transcranial Doppler (TCD) investigations for the detection of large vessel vasospasm are suggested, as well as monitoring of brain tissue oxygenation (PbtO_2_) [24, 25]. In addition, surveillance of brain energy metabolism using cerebral microdialysis (CMD) is gaining increasing interest [9, 26].

CMD is an invasive neuromonitoring bedside technique using a catheter with a semipermeable membrane probe placed in brain parenchyma, typically in the frontal lobe. By pumping a fluid, the perfusate, that mimics cerebrospinal fluid, the CMD membrane will allow small molecules such as lactate, pyruvate, and glucose to enter the perfusate and to be analyzed at fixed time intervals. Because the returning perfusate, named dialysate, mimics the extracellular fluid of the brain parenchyma, changes in dialysate concentration of selected molecules reflects alterations of brain physiology [27, 28]. Lactate and pyruvate are used to calculate the lactate/pyruvate ratio (LPR), the best indicator of metabolic stress, and as a ratio, LPR is independent of the relative recovery of the individual molecules [26, 29].

CMD can detect energy metabolic changes up to 16 h prior to DCI, potentially enabling the clinician to provide interventions [30, 31]. To date, CMD has mainly been applied as a research instrument. However, the latest CMD consensus statement concluded that the future research should focus on the clinical applications regarding decision making and treatment regimes in the day-to-day care of patients [26].

Since late 2017, our local clinical protocols have been refined to apply the CMD surveillance data for real-time clinical decision making as a part of the multimodal neuromonitoring of patients with SAH. Hourly measurements of CMD LPR and glucose are performed and an LPR warning system with a 24/7 protocol for potential active interventions to be applied at elevated CMD LPR levels has been implemented. The aim of this study was to evaluate the use and clinical efficacy of CMD-based interventions in the NCC of patients with aneurysmal SAH and to assess the prevalence of DCI-related infarction.

## Materials and Methods

### Ethical Statement

The study was approved by the ethical review board in Lund (2017/4069) and conducted in accordance with the ethical standards given in the Helsinki Declaration.

### Patient Selection and SAH Management

This retrospective study included all patients with aneurysmal SAH monitored with CMD and treated in the NCC unit at Skåne University Hospital, Lund, Sweden between January 2018, and December 2020. This 3-year period was chosen to evaluate the recently implemented protocol for CMD-based interventions, based on the expected yearly number of patients with SAH treated in our unit. According to local protocols, patients with a Glasgow Coma Scale (GCS) motor score ≤ 5 or with an imminent risk of deterioration due to hydrocephalus received an external ventricular drainage (EVD) for ICP monitoring and cerebrospinal fluid (CSF) drainage. Each patient who received an EVD also received a CMD catheter. Patients were monitored for ICP, CPP, central venous pressure and mean arterial pressure (MAP), although not autoregulatory indices such as the pressure reactivity index (PRx). For details of our vasospasm protocol, see Supplementary Figure 1.

Patients were to be kept normovolemic, normothermic and treated with oral nimodipine (primarily 60 mg q4h). The colloid of choice for volume expansion was albumin (5 or 20%), and crystalloid (usually NaCl) was used to correct fluid deficits. Blood glucose was to be maintained between 5 and 10 mmol/L. Hb ≤ 8 g/dL, or when vasospasm was suspected Hb ≤ 10 g/dL, was treated with transfusions. Blood oxygen saturation aim was > 95% and normoventilation was used. For patients on a ventilator normoventilation with PaCO_2_ at 5.3 kPa was aimed at. In the event of brain swelling, a slightly lower PaCO_2_ (moderate hyperventilation; 4.0–4.5 kPa) could be considered; in the event of vasospasm, a slightly higher PaCO_2_ could be used (up to 6.0 kPa). Low-dose dobutamine was the initial choice to enhance cardiac output, while norepinephrine was also frequently added. A cardiac output monitor was not routinely used.

Computed tomography angiography (CTA) or digital subtraction angiography (DSA) was performed to identify the cause of the SAH, and open neurosurgical clipping or endovascular coiling was typically performed within 24 h of SAH onset should an aneurysm be detected. In addition to ICP and CPP monitoring, frequent although not systematic TCD evaluations were performed.

Cerebral vasospasm was suspected if the patient (1) deteriorated neurologically (in absence of other causes such as hydrocephalus, hyponatremia, or fever); (2) if TCD measurements showed increased blood flow velocities in the middle cerebral arteries (velocities > 120 cm/s indicating moderate vasospasm and > 200 cm/s indicating severe vasospasm, increasing velocities > 50 cm/s on daily examinations, or Lindegaard index > 3 and > 6, respectively); or (3) if two consecutive CMD samples showed elevated LPRs (vide infra) (Supplementary Figure 1).

### Data Collection

Relevant patient data including age, sex, smoking habits and diagnosed hypertension were collected (Table 1). SAH staging on arrival was based on the World Federation of Neurosurgical Societies (WFNS) scale, GCS, and the modified Fisher scale [32–34]. Retrospective clinical data including CPP, MAP and CMD values were extracted from IntelliSpace Critical Care & Anesthesia (Philips, Amsterdam, Netherlands). All other patient data and information were collected from Melior (Cerner, Kansas City, MO). Computed tomography (CT), CTA, and magnetic resonance imaging (MRI) scans were reviewed in Sectra (Sectra AB, Linkoping, Sweden).

**Table 1:** Baseline data of included patients with aneurysmal SAH

Parameter	Subcategory	*n* (%)
Sex	Male	10 (20)
	Female	39 (80)
Smoking habits	Current smoker	18 (37)
	Past smoker	7 (14)
Diagnosed hypertension		23 (47)
WFNS grade	I–II	17 (35)
	III	1 (2)
	IV–V	31 (63)
GCS	13–15	18 (37)
	9–12	10 (20)
	3–8	20 (41)
Modified Fisher grade	0–I	1 (2)
	II	1 (2)
	III–IV	45 (92)
	Missing	2 (4)
Pupils	Unilateral or bilateral dilatation	8 (16)
	Sluggish/unresponsive	7 (14)
	Normal	34 (69)
Seizure		12 (24)
Focal neurological deficit		13 (27)
Mechanical ventilator after treatment		34 (69)
Aneurysm treatment	Endovascular	36 (73)
	Microsurgery	13 (27)
CMD probe placement	Right frontal lobe	40 (82)
	Left frontal lobe	6 (12)
	Bilateral	3 (6)

*CMD* cerebral microdialysis, *GCS* Glasgow Coma Scale, *SAH* subarachnoid hemorrhage, *WFNS* World Federation of Neurosurgical Societies

Patients who had CMD monitoring for ≤ 72 h were excluded. CT images obtained during the time of CMD monitoring were reviewed to determine CMD probe placement. Patients with inadequate CMD probe placement were excluded. Patients with no CT scan during days of CMD monitoring were excluded.

### Cerebral Microdialysis

A CMA-70 catheter (CMA Microdialysis, Stockholm, Sweden) with a 10 mm, 20 kDa molecular weight cut-off semipermeable membrane tip was implanted free hand via a separate burr hole placed lateral to the EVD burr hole in the nondominant (right) frontal lobe targeting the watershed area between the vascular territories of the middle and anterior cerebral arteries. In some patients treated with microsurgical clipping, the CMD was placed through the craniotomy targeting the same vascular area. Artificial CSF pumped at a rate of 0.3 μL/h was used as perfusate fluid. The fluid was collected in microvials and analyzed bedside every hour using enzymatic techniques (CMA 600 or ISCUS Microdialysis Analyzer; CMA Microdialysis). Samples collected the first 2 h after implantation were discarded.

Microdialysis raw data were collected, processed, and analyzed to calculate the LPR. Based on previous reports [29], normal CMD values are glucose 1.7 ± 0.9 mmol/L; Lactate 2.9 ± 0.9 mmol/L; Pyruvate 166 ± 47 μmol/L; LPR 23 ± 4 [29]. Glucose concentrations < 0.05 mmol/L or > 10 mmol/L, lactate concentrations < 0.1 mmol/L or > 20 mmol/L and pyruvate concentrations < 10 μmol/L or > 450 μmol/L were excluded. If either pyruvate or lactate was excluded, the entire sample (glucose, lactate, and pyruvate) at this time point was discarded. An isolated excluded glucose sample did not necessitate the exclusion of pyruvate and lactate should these be reasonable and within the range of recent samples.

A major LPR event was defined as ≥ 2 consecutive values ≥ 40. Two additional serial LPR values ≥ 40 following a major event were considered a part of the same major event if presented within 4 h. A minor LPR event was defined as ≥ 2 consecutive values ≥ 30 not fulfilling the criteria of a major LPR event. Minor events were considered as such, even if they later transitioned into major events. LPR values between 30 and 40 following a major event were not considered as separate minor events. Thus, minor events could give rise to separate major events, but major events were not considered to give rise to minor events as these values were seen as part of the LPR normalization process. LPR normalization was defined as four consecutive samples < 30 for minor, and < 40 for major events [26].

### Interventions

CMD data were analyzed continuously to identify major and minor events. At each event, it was determined whether an active or conservative approach was taken with regard to the local protocols. For any event without active intervention, it was noted whether this course was argued in the medical record and these conservative events were either termed “argued for” or “quiet.”

The CMD protocol measures to counteract DCI were in the following order: (1) to immobilize the patient in a horizontal bed position to potentially increase cerebral blood flow, to intensify neurological assessments, to control and normalize blood oxygen saturation and plasma sodium concentrations, to control volemic status and treat hypovolemia with crystalloid or colloid boluses, to control Hb and transfuse to Hb > 10 g/dL; (2) to increase CPP by 20% by either lowering the level of CSF drainage to decrease ICP, or by increasing MAP with vasopressors or inotropic drugs, preferably dobutamine; and (3) to perform CTA and based on the results consider DSA and angioplasty, for instance intraarterial verapamil administration (Supplementary Figure 1).

### Outcome Measures

The primary outcome of this study was DCI-related infarcts defined as cerebral infarcts on CT or MRI scan within 6 weeks post SAH onset, not present during the first 24–48 h after aneurysmal treatment and without apparent cause such as aneurysm occlusion [6]. Neurological deterioration alone was not considered a primary outcome. Whether the DCI-related infarcts occurred within an area monitored by microdialysis or not was noted. Each CT and MRI scan during the NCC treatment period as well as up to at least 6 weeks post injury were reviewed for the presence of cerebral infarcts by an experienced independent neuroradiologist as well as a researcher involved in the study (IH or NM).

### Statistics

Statistical analyses were performed using IBM SPSS version 26 (IBM corporation, Armonk, NY). Values are expressed as median and range or interquartile range. Patient age and day of LPR event occurrence were expressed as means and standard deviations (SD).

## Results

### Study Participants

**Fig. 1:**
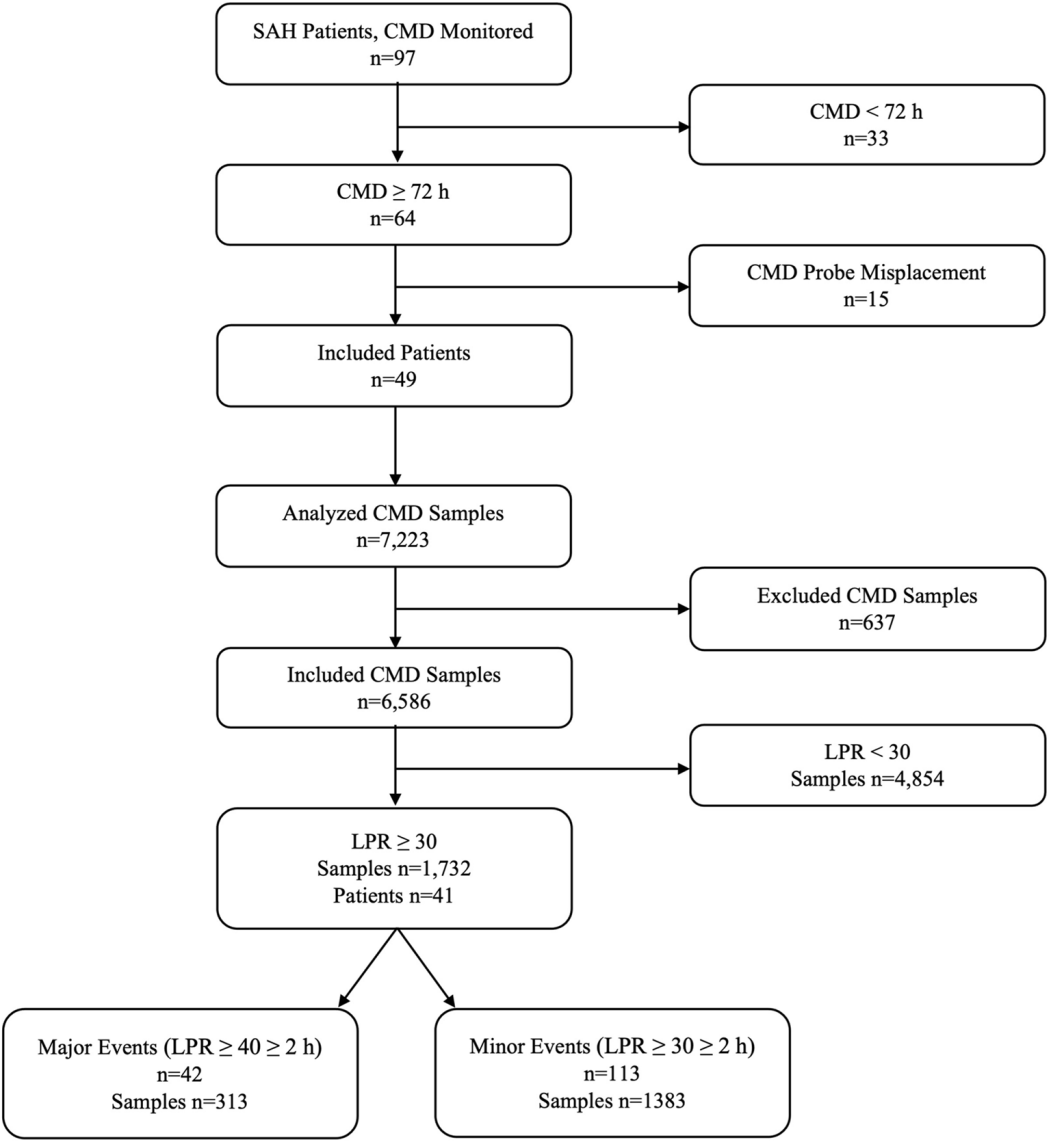
Flowchart of patients with aneurysmal subarachnoid hemorrhage (SAH) and cerebral microdialysis (CMD) samples including major/minor lactate/pyruvate ratio (LPR) events. A major CMD event was defined as an LPR ≥ 40 ≥ 2 h and a minor event was defined as an LPR ≥ 30 ≥ 2 h, not fulfilling the criteria for a major event

Between January 2018 and December 2020, 97 patients with aneurysmal SAH were monitored by CMD. Patients monitored less than 72 h (*n* = 33) or with inadequate (*n* = 8) or unknown (*n* = 7) CMD probe placement were excluded. Hence, 49 patients were included (Fig. 1). These 49 patients all had aneurysmal SAH diagnosed by CT and CTA. Most ruptured aneurysms were in the anterior circulation (82%). Baseline clinical and radiological data are shown in Table 1.

The patient group included 39 (80%) women and 10 (20%) men. The mean age was 63 years (SD 10.9). Most patients were WFNS grade IV or V (63%) on admission. Modified Fisher grade was III–IV in 45 patients (92%). Eight patients had dilated pupils and seven patients had pupils sluggish or unresponsive to light. Thirteen patients had focal neurological deficits and 12 had seizures prior to admission (Table 1).

The median (range) time from symptom onset until EVD/CMD insertion was 6 (2–36) hours. Five patients had unknown symptom onset as they were found unresponsive in their homes. Thirty-six patients received endovascular treatment, and the remaining 13 underwent craniotomy and microsurgical clipping. The median (range) time from symptom onset to aneurysm treatment was 15 (4–36) hours for coiling and 8 (2–20) hours for clipping.

The median (range) NCC admission time was 16 (4–30) days, and patients were monitored by CMD for a median of 6 (3–16) days.

### LPR

During a total monitoring time of 8,127 h, 7,223 CMD samples in 49 patients were analyzed. Of these, 637 samples were excluded. Hence, 6,586 samples could be included of which 1,734 had an LPR ≥ 30 mmol/L and 448 samples an LPR ≥ 40 mmol/L. Of the included 49 patients, 41 had 113 minor events spanning 1,277 h. All but three of these patients also had at least a single LPR value ≥ 40 mmol/L, yet only 23 patients had ≥ 2 consecutive hours of LPR ≥ 40. In total, 42 major events spanning 395 h were recorded (Fig. 1). Both major and minor events occurred most frequently on day 6 (SD 3.5) of NCC. There were more major and minor events in the higher WFNS grades (Supplementary Table 1).

At LPR elevation, ipsilateral MCA TCD values were recorded in close relation to the event in 24 major (57%) and 67 minor events (59%). The median (range) MCA flow rate was 69.5 (49–211) cm/s for major events, and 70 (26–203) cm/s for minor events. At major events, TCD values indicated vasospasm in two cases and severe vasospasm in one case based on our local protocol. At minor events, TCD values indicated vasospasm in ten cases and severe vasospasm in one case.

### CMD-Based Interventions

**Fig. 2:**
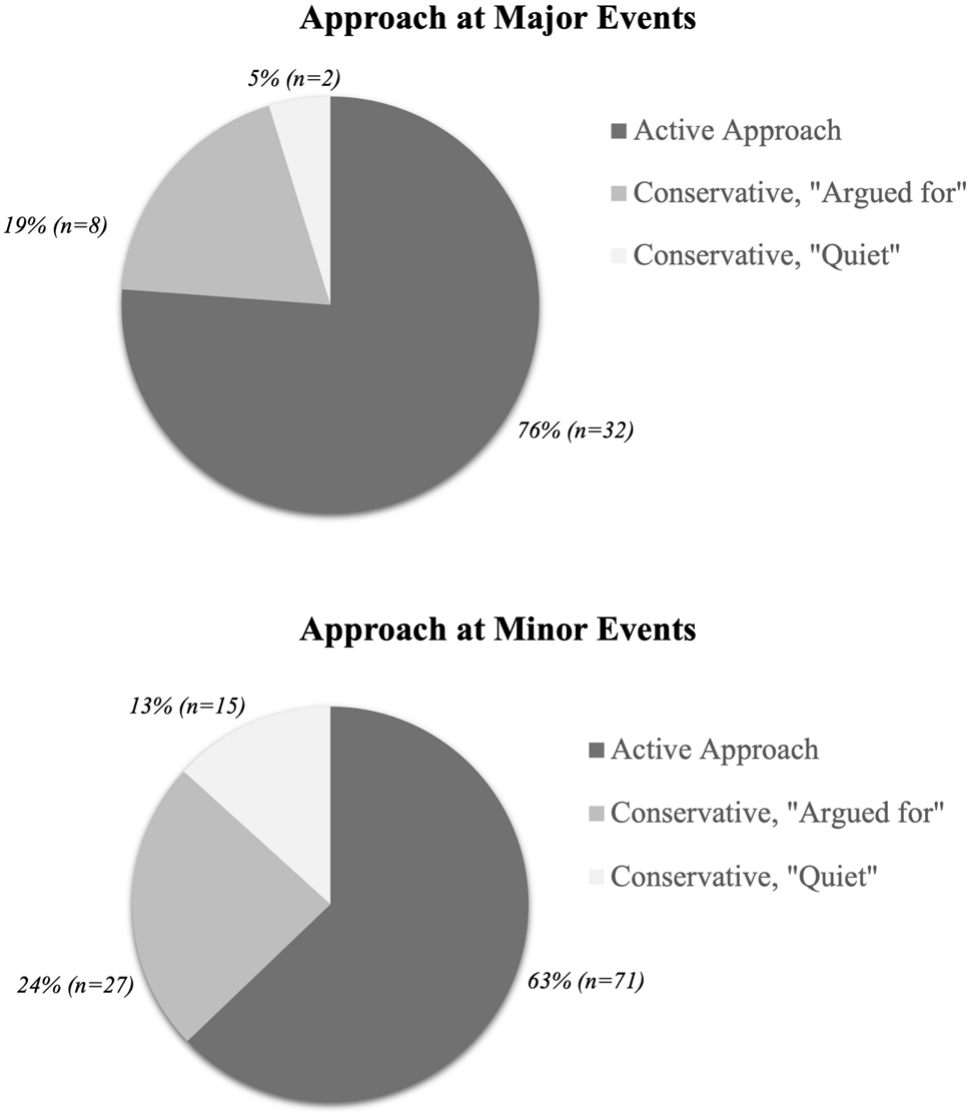
Distribution of active versus conservative approach in managing major and minor cerebral microdialysis events. A major event was defined as an lactate/pyruvate ratio (LPR) ≥ 40 ≥ 2 h and a minor event was defined as an LPR ≥ 30 ≥ 2 h, not fulfilling the criteria for a major event

An active approach was taken in 32 of 42 major events (76%). The remaining 10 events were conservatively managed without any specific intervention initiated. In eight of these, the conservative approach was argued for, whereas in two cases no note of the event was made in the medical records. At minor events, an active intervention was initiated in 71 out of the 113 events (63%). Of the 42 conservatively managed minor events, the approach was argued for in the medical records in 27 (Fig. 2). Most patients were well regulated prior to CMD events in regard to blood oxygen saturation, glucose, hemoglobin, and sodium concentrations. Of the 155 events, red blood cell transfusions were administered in 4 (Table 2).

**Table 2:** Overview of interventions at major and minor events

Parameter	Major event, *n* (%)	Minor event, *n* (%)
Nurse journal entry	28 (67)	66 (58)
Neurosurgeon or neurointensivist informed	24 (57)	46 (41)
Neurosurgeon or neurointensivist journal entry	14 (33)	23 (20)
Approach at event		
Action taken	32 (76)	71 (63)
Transfusion	2 (6)	2 (3)
Intensified mGCS/focal neurological deficit surveillance	6 (19)	21 (30)
Opened/lowered EVD setting	11 (34)	22 (31)
Vasopressors/inotropes	12 (38)	24 (34)
Colloids	16 (50)	23 (32)
Crystalloid bolus	1 (3)	6 (9)
CTA/CT preformed	6 (19)	9 (13)
Endovascular vasospasm treatment	4 (13)	7 (10)
No action, “argued for”	8 (19)	27 (24)
No action, “quiet”	2 (5)	15 (13)

Overview of active management approach, according to our treatment protocol, in minor and major events. A major CMD event was defined as an LPR ≥ 40 ≥ 2 h and a minor event was defined as an LPR ≥ 30 ≥ 2 h, not fulfilling the criteria for a major event

*CMD* cerebral microdialysis, *CT* computed tomography, *CTA* computed tomography angiography, *EVD* external ventricular drainage, *LPR* lactate/pyruvate ratio, *mGCS* motor component of the Glasgow Coma Scale

The most common action at LPR elevation was the administration of colloids, administered in 16 active major events (50%) and 23 active minor events (32%). Per protocol, an important strategy at LPR elevation was to increase CPP by at least 20%, which was the aim in 20 major events (48%). For minor events, action to actively increased CPP was taken in 39 events (35%) (Table 2).

CTA was performed at six major events diagnosing two cases of vasospasm, and at nine minor events diagnosing four cases of vasospasm. Endovascular angioplasty, some without preceding CTA, with verapamil administration was conducted at four major and seven minor events (Table 2).

### LPR Response to Interventions

**Fig. 3:**
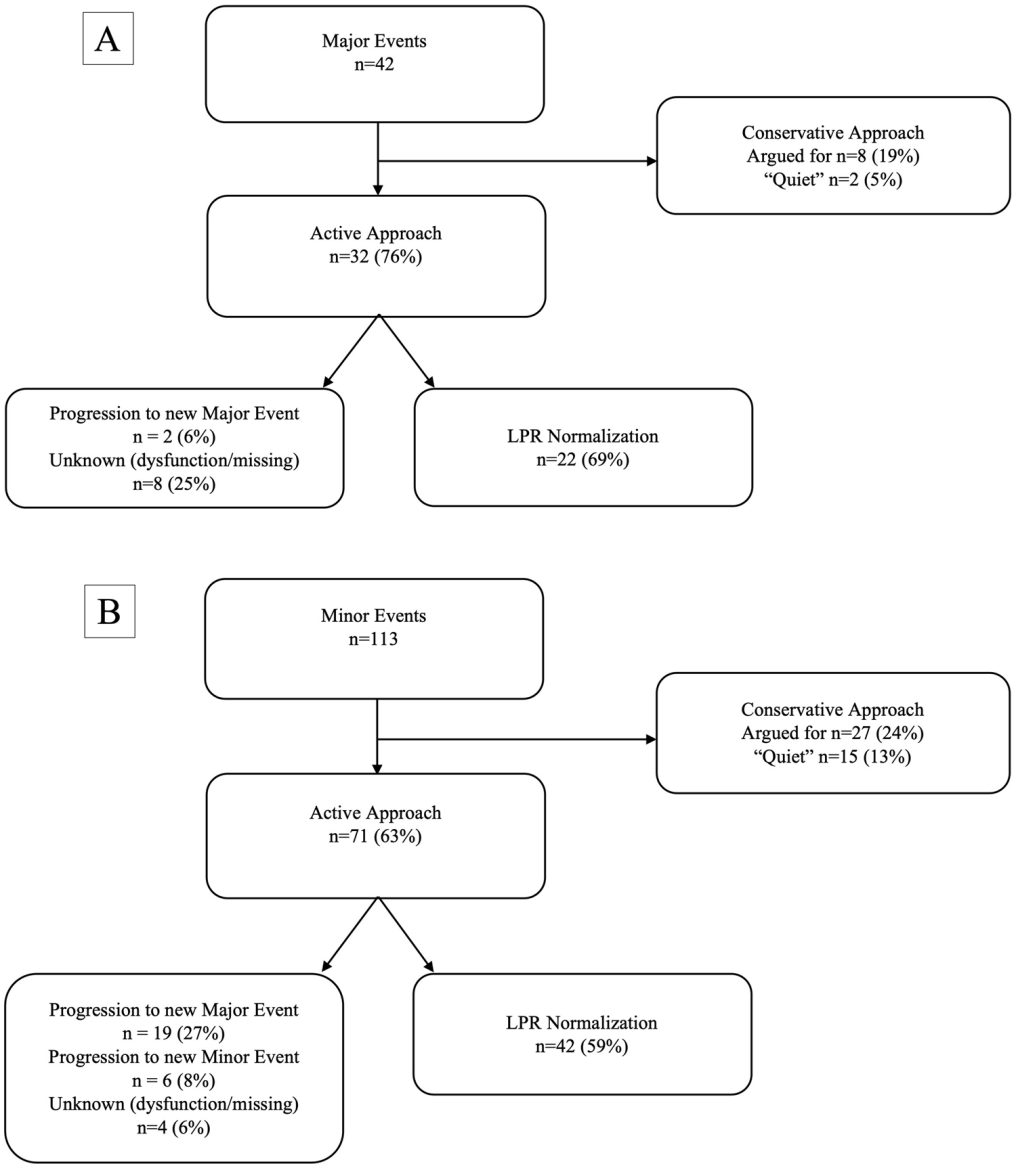
Flowchart of intervention frequency and lactate/pyruvate ratio (LPR) normalization for major (**a**) and minor events (**b**). A major cerebral microdialysis (CMD) event was defined as an LPR ≥ 40 ≥ 2 h and a minor event was defined as an LPR ≥ 30 ≥ 2 h, not fulfilling the criteria for a major event

The LPR elevation was normalized in 22 major events (69%) and 42 minor events (59%) with an active approach. In 19 cases (27%), the minor event transitioned into a major event despite active interventions. In eight active major events (25%) and four active minor events (6%) the outcome was unknown due to either missing values or CMD dysfunction (Fig. 3).

The major events managed actively had a median (interquartile range) duration of 7 (3–14) hours, whereas minor events with active approaches had a duration of 8 (3–16) hours. In contrast, the conservatively managed argued major events lasted 3 (2–5) hours and the minor event 2 (2–8) hours.

### Clinical and Radiological Outcome

New cerebral infarcts, detected by CT (*n* = 8) or MRI (*n* = 2), were diagnosed in ten patients (20%). Of these, five developed within the first day post SAH onset and/or were caused by the aneurysm-securing procedure. Thus, the total incidence of DCI-related infarcts was 10% (observed in five patients).

Infarcts developed outside the CMD-monitored area in three cases, and inside the monitored area in two. In one of these two cases, the infarct developed 6 days after CMD termination, without any infarct observed on CT scans obtained during CMD monitoring. In the other case, the patient received a new CMD probe in a brain region area with previously documented extensive vasospasm and verified infarcts on CT scan, that later aggravated (Table 3).

**Table 3:** Complications and outcome of included patients

Parameter	All patients, *n* (%)	Major & minor events, *n* (%)	Minor events, *n* (%)	No events, *n* (%)
Cerebral infarcts	10 (20)	5 (21)	4 (21)	1 (17)
DCI-related infarcts	5 (10)	3 (13)	1 (5)	1 (17)
DCI-related infarcts in CMD monitored area	2 (4)	1 (4)	1 (5)	–
3-month and 6-month survival				
Alive	43 (88)	19 (79)	19 (100)	5 (83)
Deceased	5 (10)	4 (17)	–	1 (17)
Lost to follow-up	1 (2)	1 (4)	–	–

Overview of complications during neurocritical care and survival at 3 and 6 months

*CMD* cerebral microdialysis, *DCI* delayed cerebral ischemia

The mortality rate at both 3 and 6 months was 10%, 5 out of 49 patients died. These five patients were all WFNS scale IV or V on admission and four had both major and minor LPR events during NCC.

## Discussion

In the present study, we implemented local protocols for interventions targeting CMD-based monitoring data to counteract the metabolic changes that contribute to DCI. Neurological deterioration and cerebral infarcts related to DCI are feared but potentially avoidable complications of aneurysmal SAH. The total incidence of DCI-related infarcts among the included patients in our study was low, with only two cases of infarcts in the vascular territories monitored by CMD. As addressed in several recent reviews [26, 35–37], there is a gap in the knowledge on the clinical application of CMD in routine care of patients with SAH, and its role in clinical management has not been established. The implementation of CMD in the standard care of patients with SAH requiring EVD, as suggested in our present study, may be an important transition for microdialysis from a research tool to become an integrated part of the advanced multimodal neuromonitoring.

The CMD LPR events were approached with an active intervention or argued conservative approach in a vast majority of events. Thereby, the introduced protocols resulted in CMD becoming integrated into our multimodal neuromonitoring strategy for patients with SAH, and that CMD monitoring data led to active interventions at signs of metabolic disturbance. According to the 2014 CMD consensus statement, LPR values ≥ 25 should be considered a warning sign for, and values ≥ 40 a critical cerebral energy metabolic disturbance [26]. For the purpose of this study, the lower cutoff set at 30 was considered minor events, whereas a critical LPR elevation ≥ 40 was considered major events. This view on metabolic stress was reflected in the results as major events warranted a higher degree of active intervention.

To date, the LPR is the most widely studied marker for cerebral energy metabolic stress in patients with SAH, and an elevated LPR is consistently associated with a poor outcome [26, 35]. Using CMD and LPR analyses, energy metabolic changes were detected up to 16 h prior to manifest DCI [30, 31]. Furthermore, one previous study showed CMD had higher specificity than TCD for predicting DCI development [38]. Less is known whether applying active intervention against LPR elevations may alter the course of events leading to the development of DCI. CMD is considered a safe monitoring technique and adverse events such as bleeding and infection are rare [38, 39].

Only a proportion of all vasospasm results in DCI as other factors, such as collateral circulation, blood pressure and metabolic demands influence the degree of cell damage [11, 12]. To prevent DCI, patients with SAH are kept under close observation with regards to fluid balance, electrolyte disturbances, blood oxygen saturation and CPP levels [40]. Although many management strategies have been evaluated, few have been shown to be effective in preventing DCI development [13]. Thereby, effective primary monitoring to detect early signs of DCI is vital. With evidence pointing to a complex, multifactorial cause of DCI, a multimodal neuromonitoring approach is needed. This is especially evident in patients with poor-grade SAH because they require sedation and mechanical ventilation which makes neurological assessments difficult [9, 23]. CMD measures the metabolic state of the parenchyma in real time, at bedside, and can identify signs of disturbance even in other causes than large vessel vasospasm [31]. CMD should be regarded as a complement to, rather than a replacement of, other monitoring modalities.

“Triple H” therapy, based on hypervolemia, induced hypertension and hemodilution, was long standard therapy for SAH patients [41]. However, based on the lack of robust evidence, more recent studies have advocated euvolemia and induced hypertension in selected patients [25, 42]. Because cerebral hypoperfusion at a CPP ≤ 70 mm Hg has been associated with increased episodes of energy metabolic distress and LPR ≥ 40 [43, 44], one important measure in our protocol was to increase CPP by 20%, either through lowered EVD settings or elevations of MAP [12]. Euvolemia was kept with active management of volume status. Overall, our protocol targets several potential active measures that, in combination, may act to reverse the cerebral energy metabolic distress.

The insertion of CMD catheters was done in conjunction with EVD implantations shortly after patient arrival. Patients had CMD monitoring for a median of 6 days with probe dysfunction being the reason for termination in 71% of cases. This means that some patients were without CMD monitoring during the final days of the vasospasm phase [45]. One patient developed DCI in the monitored area 6 days after CMD dysfunction. CMD monitoring extending throughout the period of peak incidence of vasospasm would plausibly be of benefit.

It is important to emphasize that CMD is a focal monitoring tool, and that only secondary insults occurring within the monitored area can be identified to trigger potential interventions. In patients with diffuse SAH bleeds, the CMD probe was placed at the right frontal lobe with the exception of patients receiving craniotomy for aneurysm clipping. We aimed at addressing the limitation of focal monitoring by placing the CMD probe in the watershed area between two vascular territories. In a previous study, CMD monitoring was unable to indicate infarcts in the contralateral hemisphere or distant ipsilateral brain regions more than 4 cm away [30]. Thus, we may have been unable to detect changes occurring in the contralateral (left) hemisphere. Furthermore, CMD is an invasive technique, and the insertion may per se induce changes in the surrounding brain tissue [46], why the first hour(s) of perfusate is usually discarded [26, 35].

DCI-related infarcts developed in five patients, although only one of those was observed during CMD surveillance in the monitored area. In this case, the patient experienced severe vasospasm and CMD was inserted into a region with already manifest cerebral infarcts. Despite intensive vasospasm treatment that included five intraarterial verapamil administrations, additional DCI-related infarcts were observed. As stated above, one additional patient experienced infarcts in a CMD-monitored region. However, this infarct was not present on CT scans obtained at CMD termination, rather it was diagnosed 6 days after CMD removal.

For the purpose of this study, we applied the definition for DCI as proposed by Vergouwen et al. [6]. Because clinical deterioration can be subjective and difficult to define, cerebral infarction might be a better outcome measure to be used in studies [6]. Accordingly, in our present study the prevalence of DCI was based on tissue outcome on CT and MR scans identifying cerebral infarction within 6 weeks, not present during the first 24–48 h after aneurysmal treatment and without apparent cause such as surgical aneurysm occlusion [6].

With a limited number of patients with SAH, firm conclusions cannot be drawn on whether active management based on CMD could prevent the development of DCI. However, a low DCI incidence (10%) was observed, considerably lower than in other reports. A recent meta-analysis reported that the risk of DCI development in modified Fisher grade III and IV patients with SAH was 30% and 42%, respectively [47]. In a previous randomized trial from our group comparing dosing regimens of nimodipine in SAH, a DCI incidence of 29% was observed [48]. As many studies incorporated in the meta-analysis [47], as well as our previous study [48], used different definitions of DCI, including neurological deterioration alone, the numbers cannot be directly compared. However, other studies based on a similar definition of tissue outcome as in our present study found an approximately 20–25% incidence of delayed infarcts [49–51]. One recent study compared patient outcome before and after the introduction of CMD and brain tissue oxygenation monitoring in NCC and observed a reduction in DCI-related infarcts from 45 to 22% [52]. Although the patient cohorts may be different, most of the patients with SAH in our study had modified Fisher grade III or IV, yet less than half the number of DCI-related infarcts was reported.

According to the protocols, after ≥ 2 consecutive hours of increased LPR values an active approach should be initiated, which was performed in a majority of events. Only active measures recorded in the medical record were considered. In the critical care record system that was used, infusion pumps, ventilator settings and vital parameters were logged automatically every hour. In addition, NCC nurses and assistant nurses manually registered other clinical findings of importance in defined intervals. Thus, the collected data should be considered complete regarding both action taken and time of action. We argue that the microdialysis LPR must be interpreted considering the full clinical context and other monitoring parameters. In an otherwise clinically stable, conscious SAH patient with an isolated LPR elevation and no sign of neurological deficit or deterioration on other neuromonitoring, it may be justified to closely observe, without other active treatment measures. Thus, a conservative approach might be advocated and argued for in the medical journal. However, firm conclusions of the undocumented “quiet” LPR events cannot be drawn. The lack of initiated interventions and documentation for an LPR event could either be due to the LPR event being missed, or that the event was noted, and a conservative approach was chosen on the basis of clinical evaluation without being documented.

### Limitations

The study has some limitations. The CMD only monitors a limited area of the brain tissue at risk, and in some patients the CMD monitoring time may have been too short to enable the detection of delayed cerebral energy metabolic changes. Thus, the frequent occurrence of CMD probe dysfunction resulting in gaps in the monitoring for secondary insults in some patients is a limitation. The cause of the CMD dysfunction is at present unknown. However, we have attempted to prevent dysfunction by refining the surgical implantation technique.

Although positron emission tomography may have provided more details to the extent of energy metabolic perturbation [53], it was not performed here. On the other hand, repeated positron emission tomography studies are rarely performed and CMD may be the best monitoring tool to assess continuous energy metabolic changes [6, 30, 31]. Furthermore, PbtO_2_ monitoring is not routinely performed in patients with SAH at our institution, and is not included in our current protocol. Arguably, several reports have suggested an important role for PbtO_2_ in the multimodal neuromonitoring of patients with SAH. PbtO_2_ monitoring could have aided in differentiating ischemic from nonischemic causes of energy disturbance. However, here we used the increased LP ratio as an indicator of energy metabolic disturbance without differentiating between a failure of oxygen delivery (ischemic hypoxia) or mitochondrial dysfunction [26].

ICP is in our department most commonly monitored using a combined intraventricular and intraparenchymatous monitor, ensuring correct ICP readings despite the EVD being opened for CSF drainage. By CSF drainage, intracranial hypertension is uncommon in these patients. Although CMD and ICP were not correlated in the present study, a contribution of ICP crises to our present findings is unlikely. At the time of many LPR events, TCD investigations were not available, nor were CT-perfusion imaging studies [54], and we cannot directly correlate the LPR events to routine bedside assessment of large vessel vasopasm and brain perfusion. Although TCD investigations were not systematically performed, our data argue for an independent role of CMD monitoring with LPR events occurring despite relatively normal TCD readings. The interventional measures taken were at the discretion of the treating physician, which may have contributed to different interventions initiated or that conservative approaches were selected.

## Conclusions

The study focused on CMD as a well-incorporated part of the clinical decision making and multimodal monitoring in patients with SAH. Although only 10% of the patients included in the study developed DCI-related infarcts, LPR events indicating energy metabolic disturbance of the monitored brain tissue were commonly observed. Adherence to the new local protocols may be associated with a decreased DCI incidence by promoting active management and investigations at time of LPR events. Future studies in larger cohorts of patients with SAH are needed to validate these findings.

## Supplementary Information

Below is the link to the electronic supplementary material.

Supplementary file1 (DOCX 69 kb)

Supplementary file2 (DOCX 13 kb)
