# Supplementary material for: Cerebral Microdialysis-Based Interventions Targeting Delayed Cerebral Ischemia Following Aneurysmal Subarachnoid Hemorrhage
Source: Neurocrit Care. 2022 Apr 29;37(1):255–66. doi: 10.1007/s12028-022-01492-5 (PMC9283139; doi:10.1007/s12028-022-01492-5)
Supplement: Supplementary file 1 — Supplementary file1 (DOCX 69 kb) [file 12028_2022_1492_MOESM1_ESM.docx]

**Protocol for management of vasospasm/delayed ischemic deterioration**

**Suspicion should be raised if:**

Altered clinical status - Neurological deterioration with onset or worsening of focal neurological deficit and / or decreased level of consciousness indicate vasospasm.

Transcranial Doppler – Blood flow velocities in MCA above 120 cm/s are associated with moderate vasospasm, velocities above 200 cm/s are associated with severe vasospasm. An increase in blood flow velocities of over 50 cm/s in one day and the Lindegaard index > 3 is also associated with vasospasm.

Cerebral Microdialysis - 2 consecutive lactate-pyruvate ratios (LPR), above the value stated in the daily treatment plan, suggest cerebral energy metabolic disturbance, and indicate vasospasm. The value for LPR is individual, but pathological values ​​are usually above 25.

**Treatment:**

Measures are to be taken in the following order. Only as many as needed to reverse symptoms or LPR elevations.


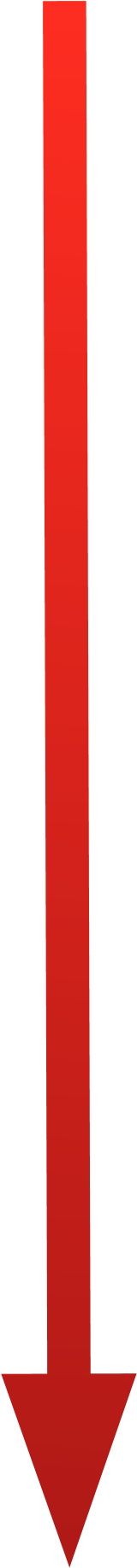


1.

Immobilization / flat-bed position

Increase frequency of neurological assessments

Check hemoglobin count, transfuse if Hb <10g/L

Check blood oxygen saturation, should be >95%, add O2 if needed

Check fluid status, should be euvolemic, treat hypovolemia

Check and correct Na - treat hyponatremia

**1000 ml NaCl 0,9% with**

**80-150**

**mmol Addex Na (during 18-24 h)**

with guidance of U Na/K.

Consider Fludrocortisone (Florinef).

2

.

Take measures to increase CPP, by lowering ICP (if possible, by lowering ventricular catheter drainage levels) and raising MAP (with dobutamine as first choice).

- overall, the measures should lead to an increase in CPP by 20%

3.

Angiography and consideration to perform endovascular angioplasty

**Supplementary Figure 1.** The protocol implemented in our department to manage vasospasm and delayed cerebral deterioration with active interventions.
