# Supplementary material for: Cerebral Microdialysis-Based Interventions Targeting Delayed Cerebral Ischemia Following Aneurysmal Subarachnoid Hemorrhage
Source: Neurocrit Care. 2022 Apr 29;37(1):255–66. doi: 10.1007/s12028-022-01492-5 (PMC9283139; doi:10.1007/s12028-022-01492-5)
Supplement: Supplementary file 2 — Supplementary file2 (DOCX 13 kb) [file 12028_2022_1492_MOESM2_ESM.docx]

## Major and minor events separated by WFNS subgroup

|  | WFNS I-II | WFNS III | WFNS IV-V | Total |
| --- | --- | --- | --- | --- |
|  | n (%) | n (%) | n (%) | n (%) |
| Major Event | 15 (36%) | 0 (0%) | 27 (64%) | 42 (100%) |
| Minor Event | 43 (38%) | 3 (3%) | 67 (59%) | 113 (100%) |
| Major and Minor Events | 58 (37%) | 3 (2%) | 94 (61%) | 155 (100%) |

**Supplementary table 1.** The distribution of major and minor events between the World Federation of Neurosurgical Societies (WFNS) scale subgroups. A major cerebral microdialysis (CMD) event was defined as a lactate/pyruvate ratio (LPR) ≥ 40 ≥ 2 h and a minor event as an LPR ≥ 30 ≥ 2 h, not fulfilling the criteria for a major event.
